# Characterization of Fibers Prepared by Centrifugal Spinning from Biotechnologically Derived Chicken Gelatin

**DOI:** 10.3390/foods13162630

**Published:** 2024-08-22

**Authors:** Jakub Martinek, Pavel Mokrejš, Jana Pavlačková, Martina Hřibová, Pavel Pokorný, Dagmar Janáčová, Robert Gál

**Affiliations:** 1 Department of Polymer Engineering, Faculty of Technology, Tomas Bata University in Zlín, Vavrečkova 5669, 760 01 Zlín, Czech Republic; j_martinek@utb.cz (J.M.); mokrejs@utb.cz (P.M.); mhribova@utb.cz (M.H.); 2 Department of Fat, Surfactant and Cosmetics Technology, Faculty of Technology, Tomas Bata University in Zlín, Vavrečkova 5669, 760 01 Zlín, Czech Republic; pavlackova@utb.cz; 3 Department of Nonwovens and Nanofibrous Materials, Faculty of Textile Engineering, Technical University of Liberec, Studentská 2, 461 17 Liberec, Czech Republic; pavel.pokorny@tul.cz; 4 Department of Processing Control and Applied Computer Science, Faculty of Applied Informatics, Tomas Bata University in Zlín, Nad Stráněmi 4511, 760 05 Zlín, Czech Republic; janacova@utb.cz; 5 Department of Food Technology, Faculty of Technology, Tomas Bata University in Zlín, Vavrečkova 5669, 760 01 Zlín, Czech Republic

**Keywords:** centrifugal spinning, chicken gelatin, enzymatic hydrolysis, functional groups, fibers, bioprinting, morphology, solubility, swelling

## Abstract

The application of biopolymer-based materials is increasing due to better sustainability and environmental protection properties. Gelatin fibers have a specific surface and high porosity, which is why their use in medicine and the food industry is being researched. This article explores the potential of centrifugal spinning to produce gelatin fibers. Gelatin for fiber preparation was obtained from a non-traditional source of collagen (chicken by-products) using a unique enzymatic process. The fiber quality was compared with those prepared from gelatins produced from traditional collagen tissues (porcine, bovine). The results showed that fibers cross-linked with glutaraldehyde vapor preserved their structure even in contact with water. Using a cross-linker controlled swelling ability and solubility while maintaining the fiber structure. On the contrary, uncross-linked gelatin fibers were water soluble due to a high surface-to-volume ratio, facilitating water penetration and dissolution. Scanning electron microscopy (SEM) provided a clearer picture of the morphology of gelatin fibers obtained by centrifugal spinning. Differences in the amount of bonding depending on the raw material used and the presence of a cross-linker were analyzed using Fourier transform infrared spectroscopy (FTIR). The overall results showed that chicken gelatin is a suitable alternative to gelatins from traditional sources and can be used for preparing food and pharmaceutical packaging and coatings, fibers, or bioprinting of 3D matrices.

## 1. Introduction

There has been a growing interest in synthesizing fibers based on natural polymers. The advantages of natural polymers include biocompatibility, hydrophilicity, and non-toxicity [1]. Although most natural polymers are of limited use due to the challenges of fiber production [2], it has been possible to prepare fibers from collagen, gelatin, chitosan, and hyaluronic acid [3,4]. The traditional sources of collagen are bovine or porcine tissues, but the use of other lesser-known raw materials, such as poultry or fish, is on the rise. The consumption of chicken meat is increasing yearly, as is the number of by-products (offal, paws, heads, or bones), which are mainly used in feed mixtures or burned. Poultry by-products contain valuable substances, such as proteins (mainly collagen), fats, and minerals, which can be further utilized [5]. Our research team developed a unique biotechnological method for extracting gelatins from poultry collagen [6]. It was proven that chicken gelatins have gelling and surface properties comparable to porcine and bovine gelatins [7,8]. By adjusting the process parameters appropriately, changes in the molecular weights of the prepared chicken gelatins can be achieved [9] that allow for not only standard applications (food, pharmaceutical, and cosmetic) but also for advanced applications (3D printing, preparation of fibers, and fiber networks).

Gelatin is a biopolymer obtained by the partial hydrolysis of collagen, the most abundant structural protein in animal connective tissues such as skin, tendons, cartilage, and bones. Before the gelatin extraction step, collagen tissues are usually treated in an acidic or alkaline way, which, using more aggressive chemicals, is not environmentally friendly but also not friendly to the source material. A suitable proteolytic enzyme is offered as an alternative method [10].

To improve gelatin’s mechanical and physical properties, it is possible to induce cross-linking, which consists of forming covalent bonds between gelatin molecules. This can be achieved in three ways: chemically, physically, and enzymatically [11]. Chemically cross-linked gels are obtained by the radical polymerization of monomers with low-molecular-weight cross-linking agents. Water-soluble polymers containing a hydroxyl group can be cross-linked with glutaraldehyde. This cross-linking occurs only at low pH and high temperature. Glutaraldehyde is also used as a cross-linking agent with polymers containing an amino group under milder conditions than polymers with an -OH group. Glutaraldehyde is a toxic compound exhibiting cell growth inhibition. Therefore, other alternatives are being developed for industrial cross-linking applications, and glutaraldehyde is gradually phased out. The EU legislation allows for an acceptable maximum limit of 2 mg of glutaraldehyde per kilogram of product [12]. The US legislation allows for the use of glutaraldehyde as a cross-linker for food purposes but does not specify the amount [13].

Physical cross-linking occurs via hydrogen or ionic bonding by exposure to UV radiation or plasma, or thermally. These bonds between chains are easily formed, but the cross-linking lacks stability due to the sol–gel transition caused by temperature, pH, or ionic strength changes. Aqueous gelatin solutions, for example, become gels when cooled but turn back into sol when the temperature is raised. Such gels are called reversible. Physical cross-linking via hydrogen bonds only occurs if the carboxyl groups are protonated, and pH is, thus, an essential factor. The advantage of thermal cross-linking is due to its simple design and the fact that it does not require additional chemicals or expensive equipment. Thermally cross-linked gelatin fibers exhibit a higher cross-linking degree than plasma-treated gelatin fibers [14]. Enzymes called transglutaminases are used for enzymatic cross-linking, which causes the formation of a cross-linked structure between free amino groups in proteins. The bonds formed by transglutaminases have a high resistance to proteolytic degradation. Enzymatic cross-linking is a suitable choice in food applications where the need for chemical cross-linking agents, as in chemical cross-linking, is eliminated. The cross-linking reaction occurs via lysine and glutamine in the collagen structure [15].

Besides cross-linking agents and plasticizers, other active substances (e.g., antioxidants or antimicrobial substances) can be added to provide additional desired properties [16], for example, essential oils (tea tree, rosemary, clove, lemon, oregano, etc.) and bioactive compounds (bee pollen, ethanol extract of propolis, dried pomegranate extract, etc.) [17,18], phenolic acids (gallic acid, p-hydroxybenzoic acid, or ferulic acid), flavonoids (catechin, flavone or quercetin) [19], and propolis extract [20]. Various organic acids, such as lactic or citric acid, influence the rheological properties [21].

Gelatins are processed into final products using various techniques. Generally, these are solvent processing methods (e.g., dipping, casting, spraying) and methods with the presence of plasticizers (e.g., extrusion molding, injection molding) [22]. A specific example is the production of soft gel gelatin capsules, in which two gelatin ribbons are first formed from casted gel masses and then passed between twin rotating dies where an active substance (medicine, food supplement, cosmetics, etc.) is injected [23]. Polymer fibers are produced in several different ways. The most well-known is electrospinning, drawing from a solution, melt-blown, and centrifugal spinning [24,25,26,27].

Electrospinning is a process that produces fibers with diameters between tens of nanometers and units of micrometers; typically, fiber diameters are in the hundreds of nanometers. With this technology, we can obtain fibers from polymer solutions and melt them from the free surface (needleless electrospinning) or a needle (needle electrospinning). Parameters such as the shape and arrangement of the electrodes and spinning collector determine the final form of the fibers. Furthermore, the properties of the spinning solution, such as its viscosity, surface tension, and electrical conductivity, are essential. Environmental conditions, such as temperature and humidity, also affect the fibers’ form [24,25]. The principle of drawing is the production of fibers from a polymer solution or melt using a micropipette with a diameter of a few micrometers. The resulting fiber is deposited on the mat’s surface, where it comes into contact with the micropipette. The method is demanding in terms of the properties of the polymer solution or melt, which are required to have good viscoelasticity and cohesion yet have minimal requirements for equipment. Meltblown is a technology in which fibers are formed by hot air flowing around spinning nozzles at high speeds. Meltblown is typical for its extensive range of possible fiber diameters. However, owing to the need to heat a large amount of air, it is also energy-consuming [26]. Centrifugal spinning is beginning to be included among spinning methods. This method is based on the principle of fiber formation using the centrifugal force that acts on the gelatin due to the high speed of rotation of the spinneret. Fibers are formed due to the action of centrifugal force, which collects on the collector [27]. Depending on the design of the spinneret, we are talking about needle and needleless spinning. The spinneret for needle spinning contains a reservoir for the spun polymer, which passes through the needles due to centrifugal force. These are located around the perimeter of the spinneret. The needleless spinneret does not contain any reservoir or holes through which the polymer solution passes. It is a disk, and due to the rapid rotation, the polymer moves from the center to its edges, and a thin polymer film is formed on the disc. The polymer moves unevenly beyond its edge by rotating the disc, forming so-called fingers. The additional solution passes through the fingers to form fibers that accumulate on the collector [28]. In many cases, it can be more cost-effective than the current technologies. This includes energy (no need to heat the air) and material consumption (solvent savings). In addition, no electrical conductivity is required from the polymer liquids. However, there are also many disadvantages, such as the relatively large diameter of the resulting fibers [29].

The current literature and industrial applications do not mention the use of poultry gelatins for advanced applications. The focus of interest is using nanofibrous materials in emergency and invasive medicine to heal damaged tissues. The highly porous network of fibrous materials increases cellular activity, which is vital for tissue engineering and wound dressing applications. These matrices protect the wound from inflammation, absorb blood, and do not cause allergic reactions [30,31]. Biopolymer fibers can be applied in food packaging as nanofiber film [32,33]. The gelatin constructs can be prepared by 3D bioprinting and used, e.g., for medical applications (artificial tissues and organs) [34,35,36]. Besides gelatin, other biopolymers, such as chitosan, also meet these characteristics [37,38].

This work aimed to prepare by centrifugal spinning uncross-linked and cross-linked fibers from chicken gelatin, and to further characterize their properties (morphology, swelling, solubility, functional groups) and compare them with the fibers prepared by the same procedure from porcine and bovine gelatins.

Chicken gelatin was made from collagen obtained from poultry by-products. A biochemical method was used for the hydrolysis of collagen, which is gentler not only on collagen but also on the environment. The tested material based on chicken gelatin is innovative not only used as an unusual source of collagen but also as a method of hydrolysis.

## 2. Materials and Methods

### 2.1. Raw Materials and Chemicals

Chicken paws (Raciola, Uherský Brod, Czech Republic); porcine gelatin (198.4 ± 2.1 Bloom); bovine gelatin (197.7 ± 1.3 Bloom) supplied by Jelínek & syn Profikoření, Ltd., Havířov, Czech Republic; 25% glutaraldehyde (Penta Ltd., Praha, Czech Republic); and Protamex^®^ endopeptidase (Novozymes, Copenhagen, Denmark) were used to pre-treat the purified collagen.

### 2.2. Appliances and Tools

**Figure 1:**
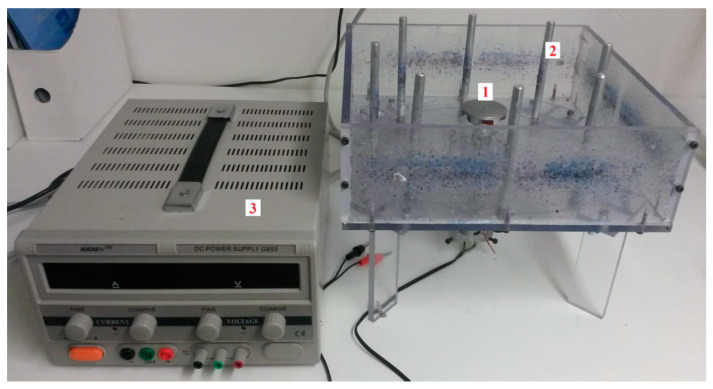
Laboratory equipment for needle-free centrifugal spinning. The device consists of a spinneret (1) with a diameter of 50 mm and a collector (2) with a diameter of 260 mm. The spinning system is connected to a laboratory power supply (3).

The needle-free spinning device was constructed at the Department of Nonwovens and Nanofibrous Materials, Faculty of Textiles, Technical University of Liberec, Czech Republic [39]; see Figure 1. It consists of a circular collector with a diameter of 260 mm and a disk spinnerette with a diameter of 50 mm. A DC motor powers the spinneret. The fiberization system is located in a polycarbonate (PC) box. The following have also been used: scanning electron microscope Phenom XL-G2 (Thermo Fisher Scientific, Brno, Czech Republic), Bruker ALPHA (Billerica, MA, USA), Scale Kern 440-49 N (Kern & Sohn GmbH, Balingen, Germany), and other laboratory equipment.

### 2.3. Processing of Chicken Paws into Gelatin

The separation of soluble proteins (albumins, globulins) from chicken paws was performed according to the procedure from a previously published work [40]. The tissue was then defatted in a 1:1 mixture of ethanol and petroleum ether [10]. The raw material was shaken with the above mixture in a ratio of 1:6 for three days, with the solvent being changed every 24 h. Gelatin extraction was carried out by a biotechnological process from chicken paws according to Patent CZ 307665—Biotechnology-based production of food gelatin from poultry by-products [6] with minor modifications. Purified collagen was mixed with distilled water in a ratio of 1:10; the pH was adjusted to 6.5–7.0. Then, the proteolytic enzyme Protamex^®^ was added in an amount of 0.4% (*w*/*w*) to the collagen dry matter, and the mixture was shaken for 15 h. Conditioned collagen was mixed with distilled water in a ratio of 1:8; gelatin was extracted at 65.0 ± 0.4 °C for four hours. The gelatin solution was dried at 55.0 ± 0.4 °C in a thin film and milled into fine particles. The characteristics of chicken gelatin from the point of view of amino acid composition are presented in Table 1. Gelatin was subsequently used to prepare the fibers.

### 2.4. Preparation of Fibres

Water gelatin solution with a volume of 20 mL, a concentration of 40.0 ± 0.3%, and a temperature of 60.0 ± 0.2 °C was gradually injected into the center of the rotating spinneret using a syringe of the same volume for 120 s. Centrifugal spinning on a needle-free device and subsequent fibers drying before removal from the collector was carried out at 21.9 ± 0.3 °C and 33.1 ± 0.2% relative humidity. Half the porcine, bovine, and chicken gelatin fiber samples were cross-linked in a desiccator with glutaraldehyde vapor for three days at 21.5 ± 0.5 °C.

**Figure 2:**
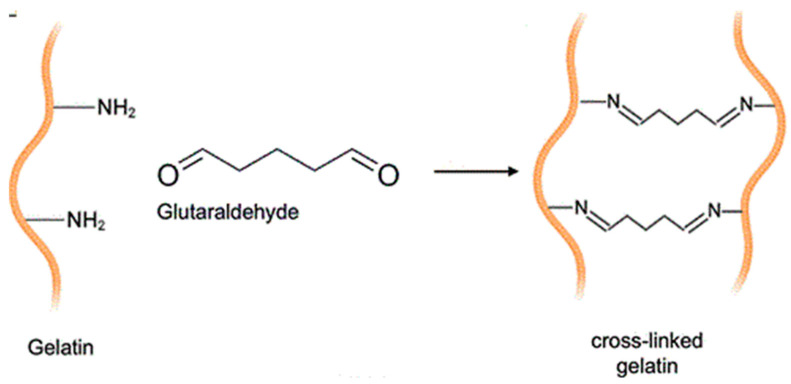
Cross-linking reaction of gelatin with glutaraldehyde.

Glutaraldehyde reacts with the amino group found in lysine, linking the two protein chains through the glutaraldehyde aliphatic chain between the nitrogen of the amino group on lysine and the nitrogen atom of the peptide bond [41]; see Figure 2.

### 2.5. Morphology of Fibers

The morphology of the fibers was observed using scanning electron microscopy (SEM). Samples were imaged using a Phenom XL-G2 scanning electron microscope at 330× magnification. The accelerating voltage used was 10 kV. For better imaging, the samples were powder-coated with a thin layer of a gold–palladium mixture.

### 2.6. Vibrational Characterization of Functional Groups

The spectra were measured for each sample using a Bruker ALPHA infrared spectroscope with Fourier transformation using the ATR method with a diamond crystal (FTIR). The samples were exposed to infrared light in the wavelength range from 400 to 4000 cm^−1^. A total of 32 images were taken during one measurement.

### 2.7. Swelling and Solubility

The experiment was carried out under similar conditions according to the method described by Padrão et al. [42]. Samples of 1.5 cm × 1.5 cm were weighed and washed with 20 mL of demineralized water for 10, 30, and 60 min, and 3, 6, 12, 24, and 48 h at 21.5 ± 0.5 °C. After being removed from the container, they were weighed again and placed in a dryer at 60.0 ± 0.6 °C for two hours and weighed again. The swelling index is determined as the quotient of the swelled sample’s weight and the sample’s initial weight. Solubility is also a dimensionless quantity given as a percentage calculated as the quotient of the weight loss between the initial and final values. Swelling and solubility were determined three times for each sample.

### 2.8. Statistical Analysis

Swelling and solubility were determined three times for each sample; arithmetic mean values and standard deviations were calculated. A one-way ANOVA was performed at a 95% significance level (*p*-value ≤ 0.05) for swelling and solubility. Tukey’s test for multiple (pair-wise) comparisons was performed; with 9 groups and 26 df, the critical Q-value for α = 0.05 was 4.3452. Statistical analysis was performed using Microsoft Office Excel 2016 (Denver, CO, USA).

## 3. Results and Discussion

### 3.1. Morphology of Fibers

**Figure 3:**
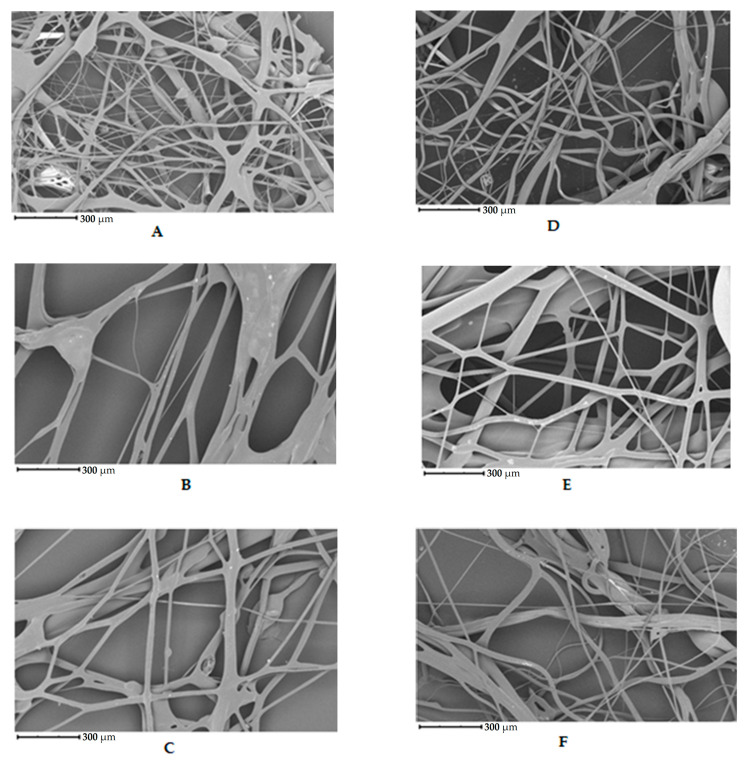
Morphology of fibers prepared from uncross-linked chicken (**A**), porcine (**B**), and bovine gelatin (**C**); cross-linked fibers prepared from chicken (**D**), porcine (**E**), and bovine gelatin (**F**).

The initial set of samples shows the structure of uncross-linked fibers from chicken, porcine, and bovine gelatin that were not further cross-linked; see Figure 3A–C. Figure 3A shows the fiber structure of chicken gelatin. The fibers form an interconnected network. The diameter of the fibers ranges from 1 to 100 µm. According to the image, the fibers are straight and curved, have different directions, and have a smooth surface. Compared to the other samples, this is the densest network, yet there are spaces between the fibers. Figure 3B shows the fibers of porcine gelatin. The fibers have a larger diameter and lower fiber density and, therefore, greater spacing than chicken gelatin. The structure is as disordered regarding the direction of the fibers as in the previous case, but the network is not so interwoven. According to the image, the surface of the fibers is smooth, and the fibers are straight, without primary curvatures. Figure 3C shows fibers of bovine gelatin. The fiber diameter is similar to that of the previous sample of porcine gelatin. According to the image, the network density is higher than porcine gelatin but noticeably lower than chicken gelatin.

The second set of samples shows chicken, porcine, and bovine gelatin fibers cross-linked with glutaraldehyde vapor; see Figure 3D–F. The fibers’ structure and diameter (1–100 µm) are similar to the previous series of samples. The fibers have different directions and are interwoven. The denser network is made up of chicken gelatin. The fibers are smooth in all cases, with a few exceptions.

The densest network of fibers was obtained from gelatin extracted from chicken collagen. The same material also produced fibers with the smallest diameter compared to the other two gelatin sources. From the images, it is evident that cross-linking does not affect the morphology observed. It is performed to improve the mechanical and physical properties of the matrix.

Gungor et al. [14] studied the effect of spinneret speed and solution concentration on gelatin nanofibers’ formation, shape, and arrangement. Gelatin fibers were prepared from a solution of bovine gelatin in acetic acid. The best quality fibers were obtained by combining a 20% gelatin solution and a rotation speed of 7000 rpm. The fibers were thermally cross-linked. Their diameter ranged from 2 to 5 µm. The fibers were smooth and straight and formed an interlaced structure without droplets. Mîndru et al. [43] observed the effect of airflow on the preparation of gelatin fibers from a mixture of acetic and formic acid. Fibers with diameters ranging from 2 to 12 µm were prepared by this method. The smooth and slightly curved fibers formed an interlaced structure without droplets. Chaochai et al. [44] prepared gelatin fibers by dry spinning them from an aqueous gelatin solution. Cross-linking was carried out using Denacol^®^ and glutaraldehyde vapor. Fiber diameters were around 60 μm. Gelatin fibers without cross-linking and cross-linked with epoxy compound Denacol^®^ had a smooth surface. Sugar-cross-linked fibers had a slightly heterogeneous surface due to the Maillard reaction, and glutaraldehyde-cross-linked fibers had a slightly rough surface. Arican et al. [45] prepared gelatin nanofiber masks by centrifugal spinning. They evaluated the morphology, porosity, and fiber diameter with the solution’s concentration and rotation speed. Fibers had an average diameter of 232–778 nm. The fibers were smooth and slightly curved, forming an interlaced structure without droplets.

The fibers prepared in this study were more significant in diameter than those from the studies mentioned above; the different preparation methods of the polymer spinning solution may have caused this. Traditionally, gelatin is spun from an acetic acid solution [14] or a mixture of acetic and formic acid [43]. In this study, it was a 40% aqueous solution of gelatin. The speed of centrifugal spinning may also influence the diameter and overall morphology of the fibers. 

### 3.2. Vibrational Characterization of Functional Groups

**Figure 4:**
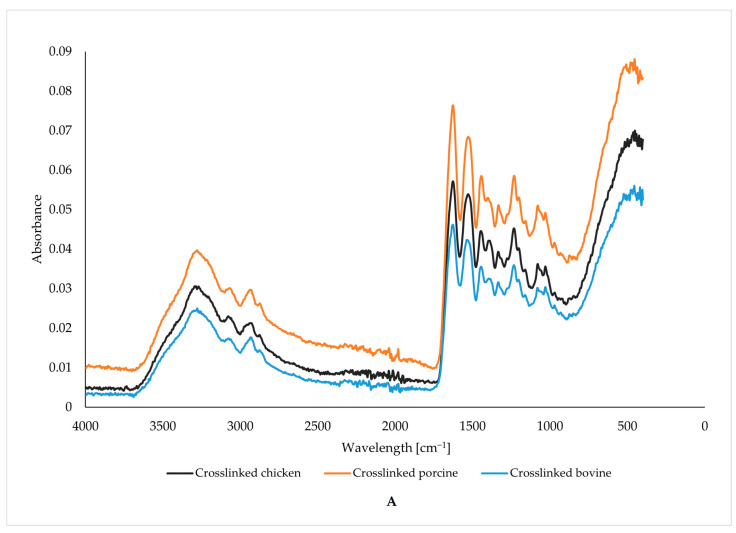

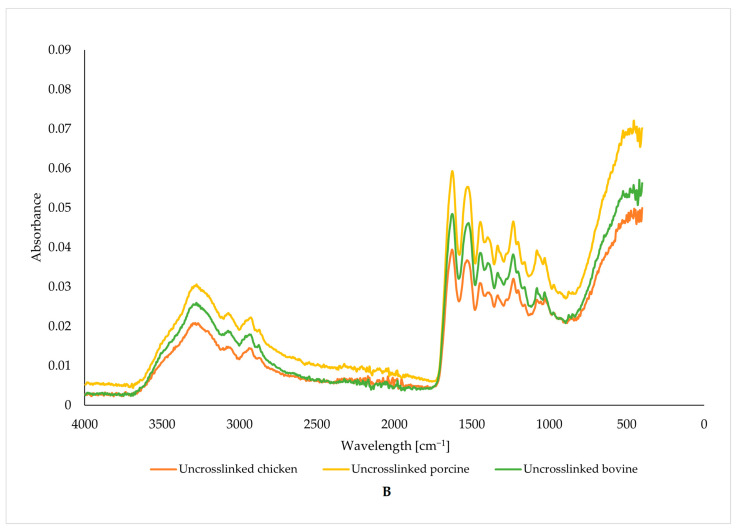
Vibrational characterization of functional groups of cross-linked (**A**) and uncross-linked (**B**) chicken, porcine, and bovine gelatin fibers.

Using the FTIR method, we can observe the effect of glutaraldehyde cross-linking on functional groups in gelatin fibers; see Figure 4. Each figure compares the spectrum of uncross-linked and cross-linked fibers depending on the origin of the gelatin.

The peaks in the spectrum of bovine gelatin fibers are located in the bands around 3278, 1628, 1537, and 1243 cm^−1^. These values correspond to amide A (stretching and oscillation of N-H), amide I (oscillation and stretching of C=O and C-N bonds), amide II (N-H bending), and amide III (bending of N-H bonds) [46,47,48,49,50]. The band around the amide I peak comprises stretching vibrations of C=O (70–85%) and C-N (10–20%) bonds. Amide I is considered the most efficient peak for protein structure analysis using infrared spectroscopy. The exact position of the amide I band depends on the hydrogen bridges and the conformation of the protein structure. In general, most proteins have multiple types of secondary structure (α-helix, β-sheet, or random structure) simultaneously, so the peak in the amide I band often shows multiple arms. The range and intensity of the amide II peak are generally much more sensitive to hydration than to changes in secondary structure [46,47,48,49,50]. 

The FTIR spectra of the bovine gelatin fibers show an increase in the intensity of all peaks when comparing the uncross-linked and cross-linked samples. However, the increase in intensity, expressed as the area under the peaks, is insignificant, as seen in Table 2. An increase in intensity, even if only slightly, implies the formation and presence of more bonds, characterized by specific wavelengths resulting from cross-linking. The graph also shows that no new peaks were formed, as the shape of the two curves is almost identical except for slightly different peak intensities.

The FTIR spectra of the porcine gelatin fibers show an increase in the intensity of all peaks comparing the uncross-linked and cross-linked samples. The increase in intensity is significant in contrast to the fibers from bovine gelatin, which can also be seen as an increase in the area under the peaks in Table 2. The increase in intensity implies the formation and presence of more bonds, characterized by specific wavelengths resulting from cross-linking. The figure also shows that no new peaks are formed here either, as the shapes of both curves are almost identical except for the different peak intensities. The increase in intensity is significant compared to bovine gelatin fibers. The increase in intensity implies the formation and presence of multiple bonds characterized by specific wavelengths resulting from cross-linking. All prepared fibers show peaks in the same wavelength regions regardless of the origin of the gelatin and the use of a cross-linker, differing only in intensity.

The peaks in the spectrum of chicken gelatin fibers are in bands around 3270, 1628, 1529, and 1232 cm^−1^. These peaks, in bands similar to those in the spectra of bovine and porcine gelatin fibers, correspond to amide A (stretching and oscillation of N-H), amide I (oscillation and stretching of C=O and C-N bonds), amide II (N-H bending), and amide III (bending of N-H bonds) [46,47,48,49,50]. The area under the peak values (see Table 2) also shows that the intensity increase is higher than the beef gelatin fibers. The increase in intensity implies the formation and presence of multiple bonds characterized by specific wavelengths resulting from cross-linking.

The figure also indicates that no new peaks were formed in this case either, as the shape of both curves is almost identical except for the different peak intensities. All prepared fibers show peaks in the same wavelength regions regardless of the origin of the gelatin and the use of a cross-linker, differing only in intensity.

When comparing the peaks of the cross-linked and uncross-linked gelatin, it is clear that there is a difference in the intensity of the selected. The highest difference in intensity was found when comparing the peaks at wavenumber 3278 cm^−1^ of the uncross-linked and cross-linked porcine gelatin; the intensity of this peak was about half that of the cross-linked sample. The higher-intensity areas under the peaks in cross-linked samples are caused by cross-linking (see Table 2). No new bonds were created, but the intensity of the existing ones increased.

### 3.3. Swelling and Solubility

In this study, both swelling and solubility are defined by weight change. When immersed in distilled water at laboratory temperature, uncross-linked bovine gelatin fibers interact with water relatively quickly, they swell, and the fiber structure breaks down. The swelling index is approximately twice that of cross-linked samples. When the sample is immersed in water, its weight increases due to water uptake, indicating an increasing swelling index. The fiber structure is stable even after subsequent drying of the sample.

The bovine gelatin fibers’ swelling values (see Table 3) were around the same value throughout the experiment. They did not increase with the time of immersion of the sample in water. The highest swelling rate was observed when the sample was immersed in water for 1 h. Conversely, the lowest weight increase occurred at the shortest contact with water. For solubility, it was similar to swelling; this parameter also did not show a significant increasing trend with increasing exposure time. The highest solubility was found when immersed in water for 3 h, while the lowest losses were observed when immersed in water for 30 min.

In contrast to bovine gelatin fibers, uncross-linked porcine gelatin fibers interact with water relatively quickly, they swell, and the fiber structure disintegrates. With time, the entire sample dissolves in the water. In this case, the solubility is the most rapid of all the samples studied. The effect of glutaraldehyde vapor cross-linking on fiber stability is noticeable. Water is absorbed when the sample interacts with water, and its weight increases. However, the structure of the fibers, unlike in the case of the uncross-linked samples, remains preserved even after subsequent drying of the sample. The swelling values increased throughout the experiment as the swelling rate increased with immersion time. The highest swelling rate was observed when the sample was immersed in water for 24 h. On the contrary, the lowest increase in weight occurred at six hours of exposure to water.

Uncross-linked chicken gelatin fibers interact with water relatively quickly, they swell, and the fiber structure disintegrates. Over time, the entire sample dissolves entirely in the water. The use of a cross-linker has a significant effect on the stability of the chicken gelatin fibers. Water is absorbed when the sample interacts with water, and its weight increases. However, the structure of the fiber is preserved even after subsequent drying of the sample. The swelling values were around the same value throughout the experiment and did not increase at about the time of interaction with water. The highest swelling rate was observed when the sample was immersed in water for 24 h. Conversely, the lowest weight increase occurred with the shortest exposure to water. For solubility, it was similar to swelling; this parameter also did not show a significant increasing trend with increasing exposure time. The highest solubility was observed when interacting with water for 10 min, while the lowest losses occurred when immersed in water for 1 h. Cross-linked chicken gelatin fibers have a stable level of swelling that does not depend on the time of immersion in water.

According to the Tukey test, there are no significant differences in swelling and solubility when comparing samples formed from cross-linked chicken, cross-linked pork, and cross-linked beef gelatin fibers. No significant differences in swelling and solubility were found even when comparing pairs of uncross-linked chicken, pork, and beef gelatin fiber samples. No significant differences in swelling were also found for cross-linked and uncross-linked samples. On the contrary, the differences in solubility were statistically significant for pairs formed from one cross-linked and the other uncross-linked sample. 

Sun et al. [51] observed swelling on fibers prepared from a mixture of gelatin and chitosan (in different ratios) with polyethylene oxide admixture. The fibers from our study showed a higher swelling index than these composite fibers. The difference may be because our fibers contained only gelatin, while the fibers from Sun et al.’s study were based on chitosan in addition to gelatin. Riyajan et al. [52] observed swelling on fibers prepared from gelatin, natural rubber, and cellulose. Fibers from the composite material from this study showed a lower swelling index than our fibers. The difference may be because the fibers were prepared from a gelatin mixture with natural rubber and cellulose. Wang et al. [53] prepared fibers from a mixture of alginate and gelatin. The swelling index increased as the amount of gelatin in the mixture increased. The swelling index of these fibers was lower than that of our fibers. Considering the above, it can be concluded that the lower swelling index is due to the presence of alginate. Padrão et al. [42] prepared fibers from fish gelatin cross-linked with glutaraldehyde vapor. In this study, they defined swelling as weight gain. Within 5 min, the fiber reached its maximum water absorption and no longer increased in weight. Their fibers’ swelling index was higher than that of our study. This may be because the source of their gelatin was fish collagen. Etxabide et al. [54] cross-linked gelatin fibers by ribose Maillard reaction; besides the influence of cross-linker concentration, they observed the effect of glycerol content as a plasticizer. Swelling values (defined as weight increase) were similar among the samples and generally higher than our results. Similar to our study, solubility was defined as weight loss compared to the original weight. The solubility values are very similar to those in our study. Uncross-linked fibers were dissolved entirely, while the dissolution loss was between 10% and 20% for cross-linked samples. Differences may flow due to a different way of cross-linking the fibers.

Using a cross-linker significantly affects the solubility and swelling of the chicken gelatin fibers. Owing to the cross-linking, the fibers are more resistant to contact with water, have a stable structure, have noticeably lower solubility, and exhibit limited swelling compared to the uncross-linked samples. Chicken gelatin fibers are less sensitive to interaction with water, which is a positive finding regarding their intended applications.

## 4. Conclusions

The novelty of the work lies in expanding the advanced application of gelatins prepared by a biotechnological method from alternative by-product sources of collagen. Fibers prepared by centrifugal spinning from chicken gelatin possess comparable properties in the observed characteristics (morphology, swelling, solubility, functional groups) as the fibers prepared from traditional gelatins (porcine and bovine). Above all, the cross-linked chicken gelatin fibers showed a higher resistance to water, which is a good prerequisite for their use in food and medicine packaging (scaffolds). Chicken gelatin has similar properties to pork or beef gelatin and can be an alternative to these traditional sources. Further research could focus on the method of physical cross-linking (UV radiation) of chicken gelatin fibers and verification of other mechanical or physico-chemical properties of the fibers.

## Figures and Tables

**Table 1:** Amino acid composition of chicken gelatin.

Amino Acid	Content [g/kg]	SD	Amino Acid	Content [g/kg]	SD
Hyp	130.36	6.51	Ile	12.45	0.62
Asp	45.03	2.12	Leu	27.45	1.23
Thr	18.06	0.89	Tyr	1.27	0.06
Ser	18.75	1.76	Phe	18.99	0.78
Glu	108.11	5.33	His	6.33	0.32
Pro	93.06	4.28	Lys	29.46	1.43
Gly	156.45	7.57	Arg	64.58	3.09
Ala	78.87	3.86	Cys	1.17	0.05
Val	17.43	0.81	Met	8.41	0.31

**Table 2:** Values are areas of significant peaks, their reference values, and the molecular action of gelatin samples.

Wavenumber [cm^−1^]	3278	1628	1537	1243
Molecular Action	Stretching and Oscillation of N-H	Oscillation and Stretching of C=O and C-N	N-H Bending	N-H Bending
**Uncross-linked gelatin**				
bovine	10.91	4.12	3.82	4.97
porcine	13.71	5.19	4.98	6.35
chicken	9.21	3.53	4.89	6.16
**Cross-linked gelatin**				
bovine	11.31	4.32	4.17	5.24
porcine	18.58	6.69	6.17	8.16
chicken	13.72	5.07	3.33	4.43

**Table 3:** Swelling and solubility of fibers from uncross-linked and cross-linked gelatins.

Uncross-linked Gelatin Fibers						
Time [h]	Swelling Index			Solubility [%]		
	Bovine	Porcine	Chicken	Bovine	Porcine	Chicken
0.17	14.3 ± 0.4	18.5 ± 0.2	13.0 ± 0.3	44.8 ± 0.3 ^a,c,e^	34.6 ± 0.4 ^a,c,e^	35.7 ± 0.5 ^a,c,e^
0.50	15.7 ± 0.2	0.0 ± 0.0	13.5 ± 0.1	54.8 ± 0.2 ^a,c,e^	100.0 ± 0.0 ^a,c,e^	47.5 ± 0.4 ^a,c,e^
1.00	0.0 ± 0.0	–	0.0 ± 0.0	100.0 ± 0.0 ^a,c,e^	–	100.0 ± 0.0 ^a,c,e^
**Crossed-linked gelatin fibers**						
0.17	5.8 ± 0.3	4.3 ± 0.1	4.2 ± 0.3	13.4 ± 0.4 ^b,d,f^	15.0 ± 0.3 ^b,d,f^	28.3 ± 0.5 ^b,d,f^
0.50	7.8 ± 0.1	8.1 ± 0.2	4.7 ± 0.2	12.4 ± 0.2 ^b,d,f^	18.2 ± 0.7 ^b,d,f^	12.8 ± 0.7 ^b,d,f^
1.00	6.3 ± 0.4	9.2 ± 0.4	5.8 ± 0.1	14.9 ± 0.4 ^b,d,f^	11.2 ± 0.6 ^b,d,f^	8.1 ± 0.4 ^b,d,f^
3	7.1 ± 0.1	6.2 ± 0.2	5.0 ± 0.3	32.8 ± 0.3 ^b,d,f^	18.5 ± 0.5 ^b,d,f^	23.3 ± 0.2 ^b,d,f^
6	6.2 ± 0.3	6.3 ± 0.2	5.2 ± 0.2	24.5 ± 0.8 ^b,d,f^	21.5 ± 0.3 ^b,d,f^	22.4 ± 0.5 ^b,d,f^
12	6.9 ± 0.2	9.5 ± 0.4	6.0 ± 0.2	18.0 ± 0.4 ^b,d,f^	11.9 ± 0.2 ^b,d,f^	27.9 ± 0.6 ^b,d,f^
24	7.7 ± 0.2	12.4 ± 0.3	6.4 ± 0.4	21.4 ± 0.3 ^b,d,f^	17.8 ± 0.5 ^b,d,f^	21.2 ± 0.5 ^b,d,f^
48	10.0 ± 0.6	11.7 ± 0.1	5.0 ± 0.3	23.9 ± 0.7 ^b,d,f^	27.7 ± 0.4 ^b,d,f^	21.3 ± 0.4 ^b,d,f^

The letters in superscripts indicate statistically significant differences between tested samples (^a^ = bovine cross-linked bovine, ^b^ = uncross-linked bovine, ^c^ = cross-linked porcine, ^d^ = uncross-linked porcine, ^e^ = cross-linked chicken, ^f^ = uncross-linked chicken) according to Tukey’s test.

## Data Availability

The original contributions presented in the study are included in the article, further inquiries can be directed to the corresponding author.
